# Wide-Field Functional Microscopy of Peripheral Nerve Injury and Regeneration

**DOI:** 10.1038/s41598-018-32346-w

**Published:** 2018-09-18

**Authors:** Ahhyun S. Nam, Jeena M. Easow, Isabel Chico-Calero, Martin Villiger, Jonathan Welt, Gregory H. Borschel, Jonathan M. Winograd, Mark A. Randolph, Robert W. Redmond, Benjamin J. Vakoc

**Affiliations:** 1 Wellman Center for Photomedicine, Massachusetts General Hospital, 40 Blossom St., Boston, Massachusetts 02114 USA; 2 Harvard Medical School, Boston, Massachusetts 02115 USA; 3 Department of Mechanical Engineering, Massachusetts Institute of Technology, Cambridge, Massachusetts 02139 USA; 4 Division of Plastic Surgery, Department of Surgery, Massachusetts General Hospital, Boston, Massachusetts 02114 USA; 5 The Hospital for Sick Children, 555 University Avenue, Toronto, Ontario M5G 1X8 Canada; 6 Division of Health Sciences & Technology (HST), Massachusetts Institute of Technology, Cambridge, Massachusetts 02139 USA

## Abstract

Severe peripheral nerve injuries often result in partial repair and lifelong disabilities in patients. New surgical techniques and better graft tissues are being studied to accelerate regeneration and improve functional recovery. Currently, limited tools are available to provide *in vivo* monitoring of changes in nerve physiology such as myelination and vascularization, and this has impeded the development of new therapeutic options. We have developed a wide-field and label-free functional microscopy platform based on angiographic and vectorial birefringence methods in optical coherence tomography (OCT). By incorporating the directionality of the birefringence, which was neglected in the previously reported polarization-sensitive OCT techniques for nerve imaging, vectorial birefringence contrast reveals internal nerve microanatomy and allows for quantification of local myelination with superior sensitivity. Advanced OCT angiography is applied in parallel to image the three-dimensional vascular networks within the nerve over wide-fields. Furthermore, by combining vectorial birefringence and angiography, intraneural vessels can be discriminated from those of the surrounding tissues. The technique is used to provide longitudinal imaging of myelination and revascularization in the rodent sciatic nerve model, i.e. imaged at certain sequential time-points during regeneration. The animals were exposed to either crush or transection injuries, and in the case of transection, were repaired using an autologous nerve graft or acellular nerve allograft. Such label-free functional imaging by the platform can provide new insights into the mechanisms that limit regeneration and functional recovery, and may ultimately provide intraoperative assessment in human subjects.

## Introduction

Peripheral nerve injuries (PNIs) affect an estimated 20 million Americans each year, with an annual cost to the US healthcare system estimated at $150 billion^1^. Fortunately, the peripheral nervous system has a relatively robust capacity for regeneration, and minor injuries often heal with full or nearly full functional recovery^2^. However, the current standards in clinical management of more severe injuries frequently yield unsatisfactory outcomes. These severe injuries, which include both nerve transections and extensive crush trauma, require surgery to reconnect viable portions of the nerve. In cases where the nerve ends cannot be brought into contact, autologous nerve grafts are used to bridge the gap. With graft repair, moderate to full functional recovery (i.e., grade 4 or 5) is achieved in approximately half of the patients^3^, leaving half with significant long-term functional deficits. In addition, complications from the nerve harvest surgery and donor site morbidity can be substantial^4^. To improve outcomes for patients with severe PNIs, there have been efforts to develop new surgical repair methods^5–7^ and engineered nerve conduits or acellular nerve allografts^8–12^ to avoid the use of autologous grafts for nerve gap injuries. While these efforts have identified promising strategies, as of yet none are sufficient to impact the current therapeutic options.

Imaging methods that are capable of monitoring nerve physiology over time are needed to address gaps in our understanding of the regenerative process, and to evaluate the performance of new treatment strategies *in vivo*. Confocal, multiphoton, coherent anti-Stokes Raman scattering (CARS), and stimulated Raman scattering (SRS) microscopies have been applied to the peripheral nervous system^13–19^, and provide a unique visualization of critical nerve parameters. However, because these microscopies interrogate small fields and image only to several hundred microns of depth in tissue, they do not effectively report on properties that span larger scales. This includes remyelination, a process that translates slowly from proximal to the injury through the repair to the distal nerve, and revascularization, which occurs across the full extent of the injury and or graft. Microscopic methods that operate over centimeter-scale fields and image the full thickness of the nerve are crucial to effectively reveal these regenerative processes. Optical coherence tomography (OCT) is based on unique physical principles that make it adaptable to larger fields^20^. While OCT has been applied to peripheral nerve imaging across expanded imaging fields, relatively poor contrast for nerve parameters has limited its utility. The microanatomy of a healthy peripheral nerve was studied using structural OCT^21^, but it was unable to measure myelination or perfusion properties. Scalar polarization-sensitive (PS) methods in OCT were then explored to measure the birefringence as a marker of myelination^22^. While promising, the high noise and prevalent artifacts in scalar OCT methods confound interpretation of the images. In addition, angiographic techniques in OCT, which have developed rapidly in recent years, have not been applied to the peripheral nerve setting despite evidence that revascularization is a critical to regeneration^23–26^, particularly in the setting of nerve grafts.

We report an OCT-based imaging platform that integrates angiographic and vectorial birefringence methods and that achieves high-contrast for both myelination and vascularization in healthy, injured, and regenerating nerves across multi-centimeter fields. In contrast to prior investigations of OCT in this area, the use of vectorial rather than scalar birefringence methods dramatically enhances both the anatomical contrast by differentiated birefringence signals from myelin from those originated from axonal and collagen organization, and allows much more detailed measures of changes in myelin-induced birefringence. OCT angiography is shown to provide detailed three-dimensional maps of perfused microvascular networks in injured and recovering nerves without degradation by vessel permeability that plagues label-based techniques when applied to wound healing settings. Furthermore, we show that the combination of vectorial birefringence and angiography within the same platform allows intraneural vessels to be discriminated from those of the surrounding tissue, and thereby provides a clearer measure of function of the revascularized tissue. We use this platform to record longitudinal (i.e., over time) measures of nerve degeneration and regeneration processes across crush and transection/repair injuries, and across different graft types. To our knowledge, this is the first demonstration of longitudinal imaging of peripheral nerve graft revascularization at capillary resolution. The results demonstrate wide-field vectorial birefringence OCT and angiography as a powerful tool in the study of PNIs, and in the *in vivo* evaluation of novel repair procedures and graft materials.

## Results

**Figure 1:**
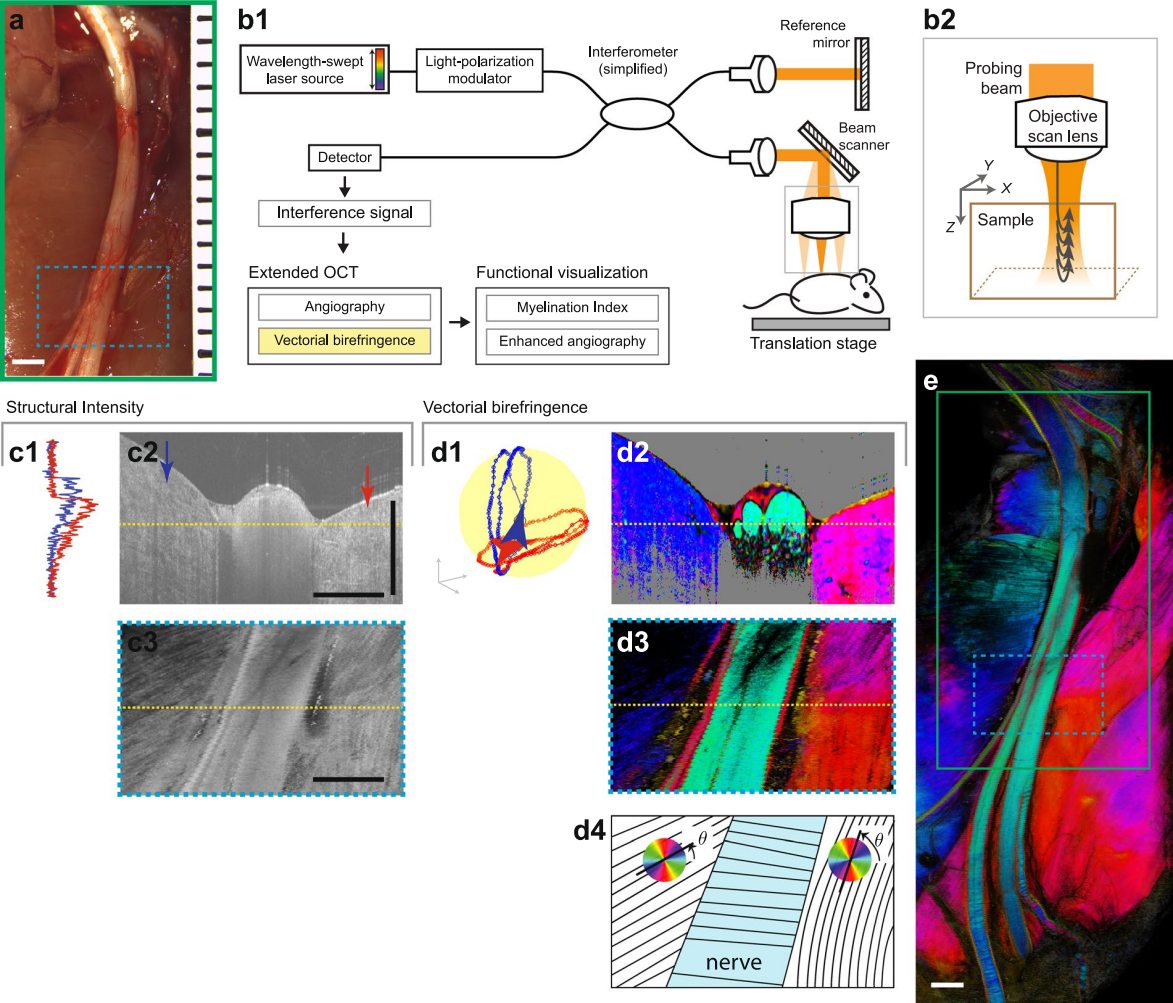
Vectorial birefringence imaging of the rat sciatic nerve microenvironment by OCT. (**a**) A photograph of the surgically exposed rat sciatic nerve. (**b**) OCT imaging system. (b1), A simplified schematic. Near infrared light from wavelength-swept source is split into sample and reference paths. (b2), Reflected signals are recorded to measure the amplitude, phase, and polarization of light reflected by each depth layer of the tissue. Volume data is acquired by scanning the probing beam in XY-plane. (**c**) Examples of structural OCT contrast, which offers only modest contrast in peripheral nerve imaging: (c1), depth-profile of the intensity at each X-position denoted by the arrows of the same color in (c2), (c2–3), tomogram (XZ) and en face (XY) cross-sections at the locations marked by the dotted yellow lines. (**d**) Examples of PS-OCT and vectorial birefringence contrast. Analyzing the state of polarization (SOP) of reflected light allows localized measurement of tissue vectorial birefringence. This information can be directly displayed using cyclic colormaps and contains information on the microstructural properties (including orientation of microfiber axis in the XY-plane) of the imaged tissues: (d1), depth-trajectory of the SOP, measured at the same locations as (c1). For simplicity, one of the two incident polarization states is displayed. (d2–3), Tomogram and en face cross-sections of vectorial birefringence equivalent to (c2–3), (d4), illustration of the optic axis orientation and display colormap. (**e**), Wide-field vectorial birefringence imaging encompassing the nerve and surrounding tissues. All scale bars = 1 mm.

The sciatic nerves of anesthetized adult rats were surgically exposed (Fig. 1a) and imaged using a custom-built vectorial birefringence OCT instrument (Fig. 1b). Imaging was performed by scanning a near-infrared light beam across the tissue and collecting the depth-resolved optical scattering signals at each location (Fig. 1b2). The amplitude, phase, and state-of-polarization (SOP) were extracted from the scattering signal and used as the basis for extracting biological parameters of the peripheral nerve. Fields as large as 1.2 cm (X-axis, faster scan perpendicular to the nerve axis) by 3.6 cm (Y-axis, slower scan parallel to the nerve axis) were captured. Details of animal models are provided in Supplementary Methods I and technical description of OCT system used for this study is provided in Supplementary Methods II.

### Vectorial birefringence imaging of the peripheral nerve microenvironment

We configured a PS-OCT instrument and signal processing methods to measure and display the three-dimensional vectorial birefringence of the imaged tissues. These birefringence measurements reveal distinct properties from those revealed by structural OCT methods. Structural OCT is based on the amplitude of backscattered light (Fig. 1c). This mode reveals microstructure based on intrinsic differences in the scattering cross-section of soft tissue. While structural OCT allows measurement of nerve cross-sectional area and gross morphology, it shows poor contrast for internal nerve structures (e.g. fascicles, epineurial and perineurial connective tissues, blood vessels). PS-OCT adds the measurement of the SOP of scattering light as a function of depth (Fig. 1d1). This provides contrast for sub-resolution ultrastructure, including collagen fiber arrangement and lipid bilayers of myelin sheaths, through their influence on the polarization of the imaging light. For example, two orthogonal polarization states of light, parallel and perpendicular to the collagen fibers, gain a relative phase difference after a forward scattering event, and this relative phase difference is accumulated in the probing beam that is a few orders of magnitude larger than the diameter of the fibers. By analyzing the trajectory of polarization across depth, local measures of the birefringence can be extracted. In biological tissues, birefringence is characterized by two parameters^27,28^: retardance and optic axis. Retardance (*ρ*) describes the magnitude of birefringence and is related to the degree of ultrastructural organization (e.g., the density of aligned collagen fibers). The optic axis defines the angular orientation (*θ*) of the birefringence (e.g., the direction of the collagen fibers). Together, the retardance and optic axis define the vectorial birefringence, i.e., the vector $\overrightarrow{B}=\rho [\cos \,\theta ,\,\sin \,\theta ]$. These measures are displayed using a cyclic colormap in cross-section or en face images (Fig. 1d2–3). In this work, these wide-field vectorial birefringence measurements provide the basis for extracting critical properties of the nerve and surrounding tissues (Fig. 1e). In comparison to the previous studies of scalar birefringence of nerve, the directional contrast added by the vectorial method provide enhanced sensitivity in low birefringence settings such as in early phase of re-myelination (Fig. S4). Detailed methods for PS-processing are discussed in Supplementary Methods III.

### Quantitative myelination imaging

**Figure 2:**
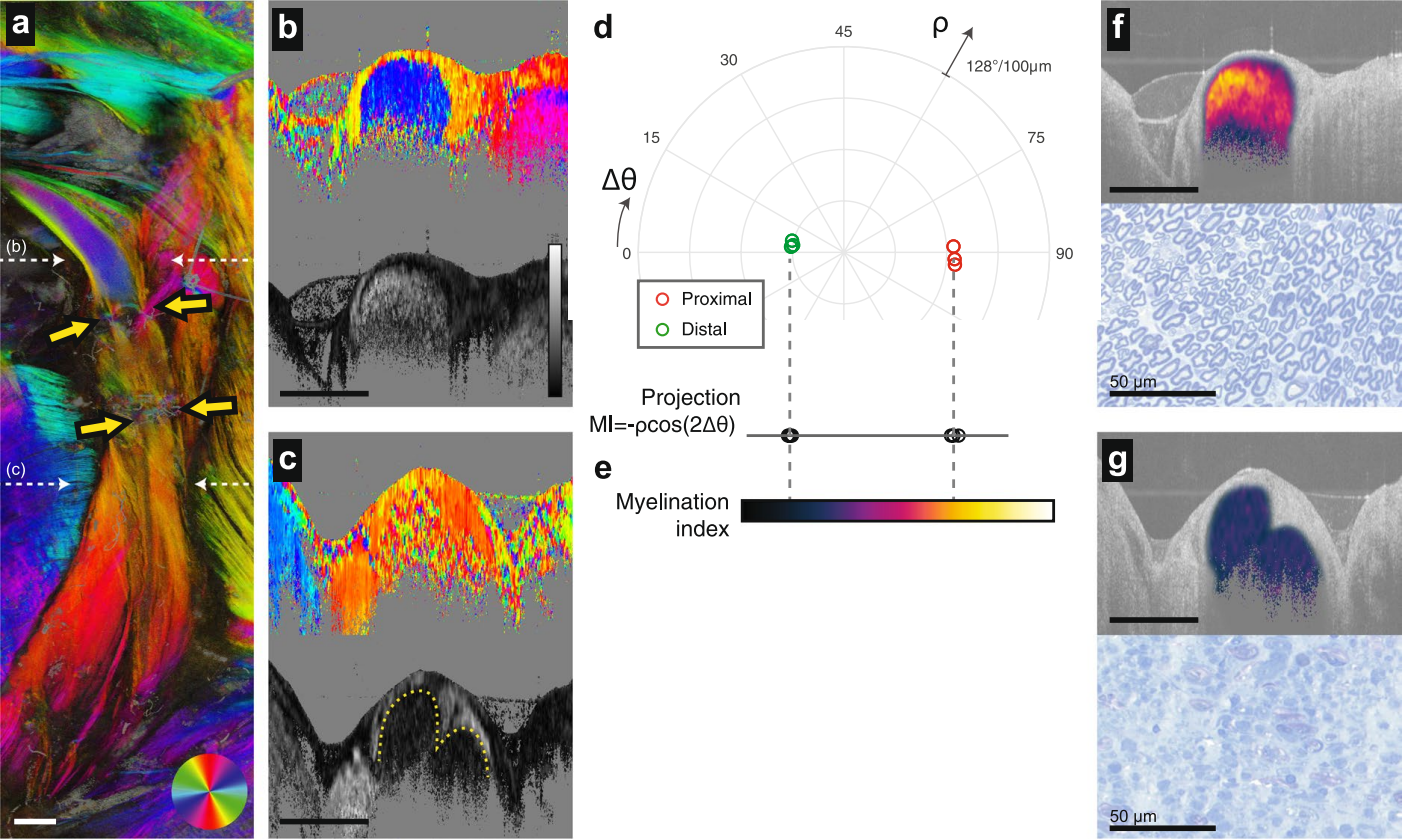
Deriving a myelination index from vectorial birefringence measurements. (**a**) Depth-projection of vectorial birefringence of the rat sciatic nerve imaged 14 days after acellular graft repair. Coaptation points are marked by the yellow arrows. (**b**,**c**) Cross-sectional visualization of vectorial birefringence (optic axis and retardance in top and bottom panels, respectively) proximal (**b**) and distal (**c**) to the graft. Imaged sites are marked by the white dotted arrows in (**a**). The solid gray backgrounds in (**b**,**c**) are applied to pixels with amplitudes below a threshold intensity. (**d**), Polar representations of vectorial birefringence of the pixels in the fascicle. Each point is obtained from complex averaging across a manually segmented cross-section. Three cross-sections are analyzed at 1.5 mm, 2 mm and 2.5 mm proximal to the suture site (red circles), and 1.5 mm, 2 mm and 2.5 mm distal to the suture site (green circles). (**e**) The MI is calculated from the projection of the vectorial birefringence to the x-axis. The colormap used to visualize MI is indicated. (**f**,**g**) A visualization of MI (calculated only for intrafascicular pixels) overlay on the structural cross-section. Image sites in (**f**,**g**) correspond to those of (**b**,**c**). In the histology images, myelin is highlighted by toluidine blue staining of nerve cross section. All length scale bars = 1 mm, unless otherwise marked. For (**b**-**c**), the grayscale colorbar scales from 0 to 128 degrees retardance per 100 micrometers.

Methods to monitor demyelinating Wallerian degeneration after injury and remyelination following repair are critical to understanding basic nerve neurobiology and to evaluating therapeutic and surgical interventions^29^. The peripheral nerve comprises various birefringent tissues including the lipid bilayers of myelin sheaths, the collagen fibers of the epineurium and perineurium, and the axons. With scalar birefringence imaging, it is difficult to distinguish between these sources to quantitatively measure myelination. In addition, high noise levels in PS-OCT are difficult to average out when using scalar methods. Vectorial birefringence imaging circumvents these limitations. Myelin induced birefringence and collagen/axonal birefringence have perpendicular orientations^16,30^, and can be distinguished by their optic axes. This provides high contrast for the internal fascicles of the peripheral nerve, and provides a means for quantifying the degree of myelination across a broad dynamic range. To demonstrate this, we present an optic axis projection of wide-field vectorial birefringence imaged 14 days after a transection injury with acellular graft repair (Fig. 2a). Here, the graft coaptation points are identified by the arrows. Proximal to the graft, the nerve remains fully myelinated (Fig. 2b), while distal native nerve has undergone full demyelination with no evidence of remyelination at this timepoint (Fig. 2c). Interestingly, in the fully demyelinated nerve, the boundary of the fascicle and external epineurium can be visualized based on the retardance (despite both tissues having the same optic axis), as highlighted by the dotted line.

The quantitative vectorial birefringence measures of the fully myelinated and demyelinated fascicle can be visualized by plotting them on a polar plot with the optic axis and retardance mapped to the angle and radius, respectively (Fig. 2d). Here, the optic axis angle is defined relative to the longitudinal axis of the nerve (rather than physical angle as in Fig. 2a–c) as angular deviation. Note the clear grouping of the measures for these two myelination states, and their perpendicular orientation in physical space (the semicircle of the polar plot spans 90 degrees of angular deviation). These measures can be projected onto the horizontal axis to create a one-dimensional value, myelination index (MI), spanning full demyelination (with a residual birefringence resulting by the combined effect of the internal epineurium, perineurium, and axons) to full myelination (with a net birefringence that is dominated by myelin) (Fig. 2e). The formula for MI is given by ${\rm{MI}}=-\,\rho \,\cos (2{\rm{\Delta }}\theta )$ where 2*Δθ* is twice the deviation angle between the physical birefringence axis and the nerve longitudinal axis, and *ρ* the retardance. The detailed definition and methods for calculating the MI are presented in the section “Computation of the MI” in Supplementary Materials. Cross-sectional images of the MI calculated within the nerve fascicle visualize the myelination properties of the tissue (Fig. 2f,g). We note here that the MI ascribes a non-zero myelination to the nerve with zero net birefringence; at this point, the myelin-induced birefringence offsets the structural (epineurium/perineurium/axons) birefringence because each is orthogonally oriented. Without vectorial measures, it would be difficult to interrogate these limited birefringence levels due to noise, and impossible to differentiate the two contributions to birefringence.

### Longitudinal imaging of demyelination and remyelination after injury

**Figure 3:**
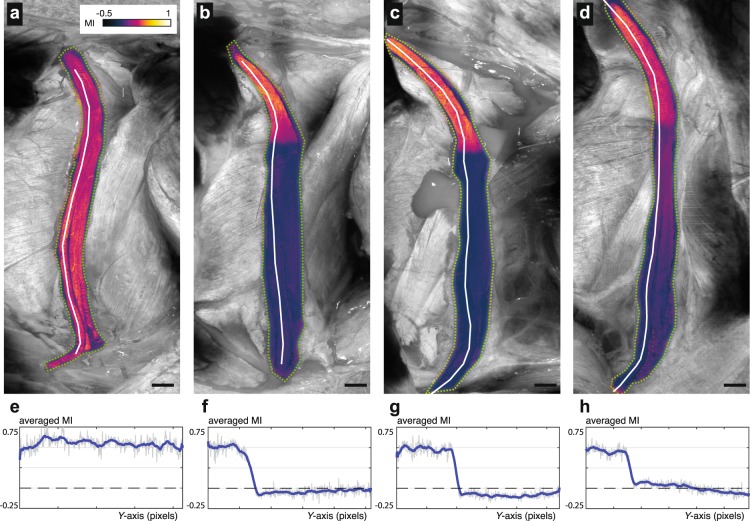
Longitudinal myelination imaging following nerve crush injury. (**a**–**d**) Myelination indices across the nerve fascicles were calculated (**a**) immediately before injury and at (**b**) 7, (**c**) 14, and (**d**) 28 days after injury. Quantitative measures of MI along the nerve longitudinal axis are plotted for the common peroneal fascicles. (**e–h**) The quantitative MI plots corresponding to the projections displayed above in (**a–d**). The values are obtained from the three-dimensional centerline in the volumetric MI data. The white lines in the projection images represent this centerline. Scale bars = 1 mm.

To demonstrate longitudinal myelination imaging, we imaged the sciatic nerve at baseline and following crush injury on 7, 14 and 28 days (Fig. 3a–d) post-injury. The myelination index was calculated throughout the nerve fascicles and projected across depth. The myelination index along the left-most fascicle, which will become the common peroneal nerve, is additionally plotted for each timepoint (Fig. 3e,f). At all timepoints, minimal changes in myelination are observed proximal to the crush injury as expected. Full demyelination distal to the injury is observed at day 14 (Fig. 3c) is in agreement with prior reports^17,31,32^. At day 28, remyelination is clearly evident in the injured nerve segments (Fig. 3d), with more pronounced remyelination near the injury site. Such changes from demyelination to remyelination at the crush site have been observed in all four animals that were subject to crush injury, as shown in Fig. S5 and discussed further in the Supplementary Methods III. The ability to image demyelination and remyelination processes across wide-fields in the same animal enables exploration of neurobiology and direct evaluation of repair procedures.

### Wide-field three-dimensional angiography with fascicular segmentation

**Figure 4:**
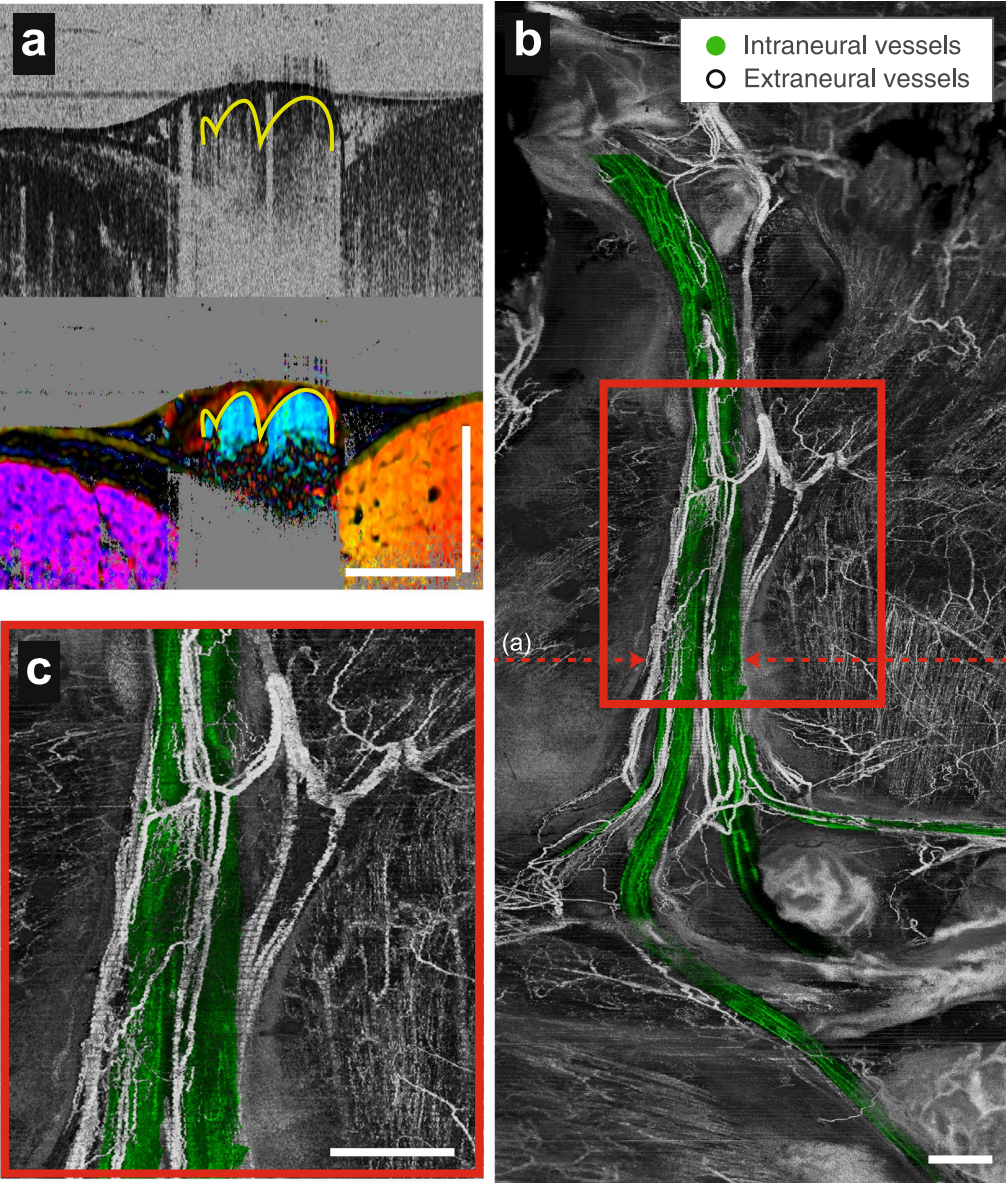
Wide-field three-dimensional angiography with fascicular segmentation in a native nerve. (**a**) Cross-sectional angiography and vectorial birefringence imaging together highlight vessels and identify those within the nerve fascicle. (**b**) Fascicular vessels are colorized green in en face angiographic projections that reveal the vascular networks in the healthy peripheral nerve and surrounding tissues. (**c**), Expanded view of the region marked by the red rectangle in (**b**). Scale bars = 1 mm.

The peripheral nerve is perfused by vessels that are oriented primarily along the longitudinal axis of the nerve and are located within the perineurium, its outer epineurium, and externally in the surrounding mesoneurium. In injury and disease, the vascular supply to the nerve can be disrupted, and timely reperfusion of the injured nerve (or nerve graft tissues) is likely a predictor of functional recovery^23,25,26^. OCT offers label-free detection of microvasculature based on time-varying characteristics of the signals from flowing blood^33,34^. We performed angiographic imaging in parallel to the vectorial birefringence imaging to provide simultaneous and precisely co-registered imaging by the two modalities. In addition, the vectorial birefringence measures were used to define the fascicles and allow the intraneural vessels to be distinguished from the vessels in the outer epineurium, the scar tissues after injury, and the mesoneurial tissues surrounding the nerve (Fig. 4a). This technique allows a more detailed understanding of the revascularization process within nerves, and provides an easy way to discriminate the vasculature in the fascicle from that in the outer epineurium and the external environment surrounding the nerve. The resulting projection image of a healthy nerve reveals the microvascular network that primarily runs in the longitudinal direction within the epineurium and smaller vessels branching into the nerve fascicles, as well as connections external to the nerve (Fig. 4b,c). The technical methods for vascular imaging are provided in Supplementary Information V.

### Longitudinal imaging of revascularization in crush and graft repairs

**Figure 5:**
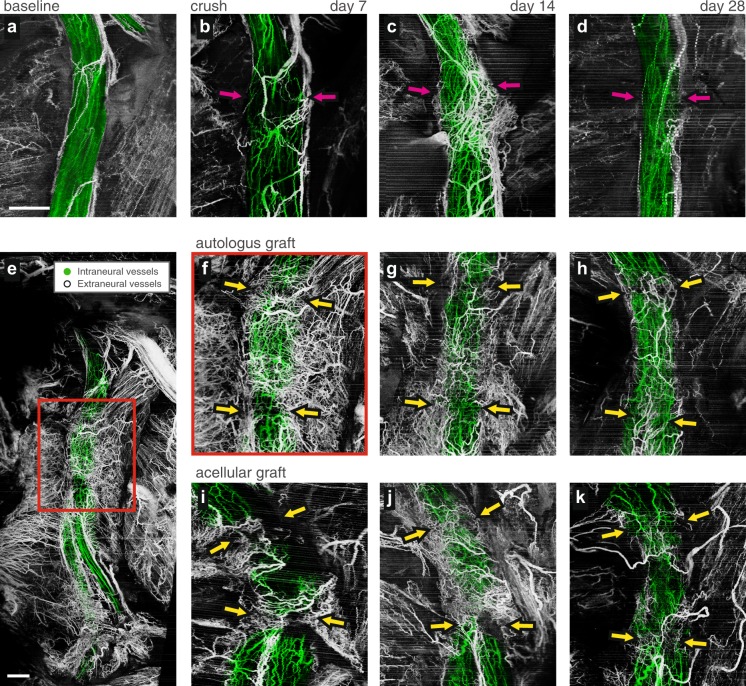
Longitudinal imaging of microvasculature following nerve injury and repair. Magnified vascular projections of angiographic OCT from a same animal (**a**), at baseline, (**b**–**d**), 7, 14, and 28 days after a crush injury, respectively. The crush injury site is marked with the magenta arrows. (**e**), Wide-field vascular projection of autologous graft acquired 7 days after surgical repair. Area marked by a red rectangle is expanded in (**f**). The projections of equivalent magnification are presented, (**f**–**h**) following autologous graft repair, and (**i–k**), acellular graft repair demonstrating longitudinal monitoring of vasculature. The images were acquired 7, 14, and 28 days after the respective surgical repair, from left to right. Coaptation points are marked with the yellow arrows. All scale bars = 1 mm, all panels except (**e**) are of the same scale.

Peripheral nerve angiography by OCT can be used to investigate revascularization in nerve repair procedures. To demonstrate this, we studied the revascularization in the first 28 days after crush injuries, and in transection injuries followed by repairs using autologous or acellular nerve grafts. Imaging was performed on the healthy nerve before injury and at 7, 14 and 28 days after injury and repair. Baseline angiography for each animal revealed similar microvascular networks showing organized and predominantly longitudinally arranged vessels (see Figs 4 and 5a). Post-injury longitudinal imaging revealed the revascularization of the graft, and highlighted pronounced differences in vascular response between the crush and transection injuries, and between the two graft repair methods. For crush tissues (Fig. 5b–d), the longitudinal vessels are mostly preserved during the recovery from injury, indicating that restoration of the vascular supply to the fascicular, epineurial and mesoneurial tissues was essentially immediate. It was also observed that additional vascular supply is provided by the surrounding tissue bed. For the autograft tissue (Fig. 5e–h), we observed a network of longitudinal vessels within the graft by day 7 both in the fascicles and the epineurium, and massive angiogenesis in the surrounding mesoneurial tissues is apparent. At this relatively early timepoint, it is expected that the reperfused vessels in the autologous graft are not *de novo* vessels. By contrast, the acellular allograft (Fig. 5i–k) shows much more limited fascicular revascularization at day 7, with a dense network of angiogenic vessels at the suture sites. In both grafts, vascular supply at the early timepoints regenerates both within the nerve and also within the surrounding tissues. By day 28, the peripheral nerve vasculature is remodeled to more closely resemble that of baseline with longitudinal vessels dominating in both graft repairs. However, even at this late timepoint, the vascular network in the autograft more closely resembled the organized networks seen at baseline.

## Discussion

By measuring the vectorial birefringence properties of the peripheral nerve microenvironment, we have demonstrated new capabilities to longitudinally characterize critical biological parameters of the peripheral nerve. In comparison to existing microscopies, the OCT methods demonstrated here offer higher speeds, deeper imaging, wider-fields, and the ability to characterize multiple parameters without transgenic modifications or exogenous labels. The latter can be a critical advantage in many experimental settings including trauma, wherein, for example, label-based angiography is degraded by extravasation due to high permeability of wound vessels. Additional advantages in traumatic settings include the ability to image more effectively through scar tissues surrounding the nerve. This allows longitudinal imaging without needing to dissect scar tissue, which would disrupt the wound healing process that is being studied. While this work is demonstrated for preclinical imaging, a key advantage of these vectorial birefringence OCT methods is their label-free nature, and their ability to translate to clinical studies. There is potential for these OCT methods to evolve to provide intraoperative^35^ or minimally invasive assessment of nerve health, and to guide the clinical care of nerve injuries. We additionally note that the peripheral nerve lipid membranes affect optical signals not only through their microstructure organization (i.e., birefringence) but also through their fundamental coupling to the membrane potential, and that these dynamics can be detected by label-free optical methods^36–39^. It may be possible to add contrast for neural activity, using OCT or another modality, to more comprehensively assess nerve physiology.

Furthermore, these OCT methods create new research opportunities to specifically study peripheral nerve injuries, and to evaluate methods to stimulate and repair damaged nerves. A critical need in this area is to develop effective methods to repair severe nerve trauma using acellular grafts, which would avoid the significant morbidity associated with harvest of graft tissues from the individual. However, these acellular grafts have not yet matched outcomes that are obtained by autologous graft in repair of injury involving large nerve deficit^1,11,12^. By elucidating the mechanisms responsible for this differential response through longitudinal imaging in the same animal, including for example the differences in revascularization of the grafts, it may be possible to improve surgical procedures and engineer better nerve graft products. Finally, it is important to further enable imaging outcome measures in preclinical studies that directly interrogate underlying mechanisms such as myelination and vascularization, which may provide predictive information on nerve regeneration in shorter time-frames than functional measures (electrophysiology, muscle strength testing, ambulation metrics) due to the long functional recovery time in large-animal models.

## Materials and Methods

### Animals

The purpose of this study was to investigate the capability of our multi-functional (angiographic and vectorial birefringence imaging) OCT platform in longitudinal assessment of peripheral nerve regeneration process. Rodent sciatic nerve model was chosen because of the similar caliber and size to the human digital nerve^21^. The OCT system and processing pipeline were developed for PS and angiographic imaging. The data acquisition protocol and microscope interface were optimized for rodent sciatic nerve imaging.

All animal procedures were approved by the Massachusetts General Hospital Institutional Subcommittee on Research Animal Care and cared for according to the National Institutes of Health Guide for the Care and Use of Laboratory Animals. Detailed of experimental procedures are provided in Supplementary Methods I. A total of 12 animals were randomly assigned to one of the three groups of n = 4: (1) crush injury, (2) transection and repair with autologous graft, and (3) transection and repair with acellular nerve graft. The imaging protocol was performed on the surgically exposed sciatic nerves. All animals were imaged at baseline (before the respective injury and repair), then 7, 14, and 28 days after the injury/repair. No anastomotic repair was performed on the crush injury group. After the last imaging session on the post-operative day 28, animals were euthanized and both sciatic nerves were harvested and processed for histology. In addition, an additional 3 animal cohort was used to correlate OCT and histology measures are different time-points. Here, the sciatic nerves were subject to a crush injury and imaged before and after the injury, and 5 days after the injury. After the last imaging, the animals were euthanized and both sciatic nerves were harvested and processed for histological analysis under toluidine blue stain.

### Instrumentation

The imaging system used for this study is developed to record simultaneous angiography and vectorial birefringence measurements using OCT. The OCT system employed a wavelength-swept laser source constructed using a polygon-mirror scanner^40^. The source operated at a 50 kHz A-line rate, and the spectrum was centered at 1310 nm, spanning a bandwidth of 130 nm. A schematic of the system is presented in Fig. S1. The basic design of the imaging system followed our previous description^33^, but with an additional electro-optic modulator in the sample arm to modulate the polarization state of the beam illuminating the sample between alternating A-lines^41^ to obtain the PS contrast free from ambiguity. In the microscope, a commercial OCT scan lens (Thorlabs LSM03) was used as the objective with a collimated beam of a 3 mm diameter, yielding a predicted transverse resolution of approximately 15 µm. The microscope and acquisition protocol were customized for optimal imaging of the rat sciatic nerve, to maintain an optimal transverse resolution over a wide field of view (10 mm in X and 24 mm in Y) that captures the entire nerve segment. Further details are provided in Supplementary Methods II.

### PS processing for vectorial birefringence analysis

Further details of processing and vectorial analysis are presented in Supplementary Methods III. This section summarizes the overall method.

The local birefringence (phase retardation *ρ* and optic axis orientation *θ*) of the tissue was first calculated using the spectral binning algorithm^41^, which mitigates the effect of polarization mode dispersion in the fiber-based OCT system by calculating the local retardation using several windows of narrow spectrum. Here, the computation is performed using Stokes formalism where the optic axis is represented by a three-dimensional vector. Then, the least-square fitting was used to reduce the dimension of this optic axis vector into its orientation in XY-plane, i.e. a one-dimensional angle *θ*. This fitting process was necessary because the optic axes plane can be rotated off the linear-polarization (QU) plane due to the birefringence of the fiber in the imaging system^42^. The optic axis orientation *θ* and formerly obtained phase retardation *ρ* are treated together as a vector quantity, and then visualized using a two-dimensional hue-value (HV) colormap. A flowchart describing this process is given in Fig. S2.

The structural alignment of lipid molecules in the myelin sheath bilayer allows for discrimination between the contribution of myelin and that of the epineurium to the measured local birefringence through the orientation of the optic axis (Fig. S3). Based on the perpendicular nature of the microanatomy, status of myelination can be quantified using MI. The centerline of the nerve is manually defined in the Z-stack projection, and the projection of the vectorial birefringence to this centerline is defined as MI. The directionality of vectorial birefringence is critical when further processing the PS-OCT measurements, as shown in Fig. S4.

### OCT Angiography

Co-registered angiography was achieved by implementing a segmented bidirectional scan pattern in X-direction with a galvanometer^43^. The scanning protocol yields 7 measurements at each of 1184 transverse locations in the X-axis. Each of the four forward scanned measurements and the three backward scanned measurements are separated by 12 ms. To generate angiographic images, we use a pairwise comparison described in our previous publication^33,34^.

We note that this angiographic algorithm includes both phase and intensity signals, but operates such that the angiographic signal is largely immune to bulk phase shifts caused by axial motion or phase artifacts resulting from asynchronous laser and digitization systems. Because of the proximity of the sciatic nerve to the body, the imaging was affected by respiratory motion. This motion in turn degraded angiographic contrast. The degradation can be reduced by simply discarding the most-affected differential pairs from the processing. Fig. S5 provides an illustrative flow diagram of the motion artifact suppression method, and the effective motion artifact reduction is demonstrated in Fig. S6. Further details of the artifact suppression and segmented projection is discussed in Supplementary Methods IV.

## Electronic supplementary material

Supplementary Materials
