# Supplementary material for: Wide-Field Functional Microscopy of Peripheral Nerve Injury and Regeneration
Source: Sci Rep. 2018 Sep 18;8:14004. doi: 10.1038/s41598-018-32346-w (PMC6143548; doi:10.1038/s41598-018-32346-w)
Supplement: Supplementary file 1 — Supplementary Materials [file 41598_2018_32346_MOESM1_ESM.pdf]

Wide-Field Functional Microscopy of Peripheral Nerve Injury and Regeneration

Ahhyun S. Nam[1,2,3†], Jeena M. Easow[1,2,4†], Isabel Chico-Calero[1,2], Martin Villiger[1,2], Jonathan Welt[1], Gregory H. Borschel[5], Jonathan M. Winograd[2,4], Mark A. Randolph[2,4], Robert W. Redmond[1,2], Benjamin J. Vakoc[1,2,6*]

[1] Wellman Center for Photomedicine, Massachusetts General Hospital, 40 Blossom St., Boston, Massachusetts 02114, USA.

[2]Harvard Medical School, Boston, Massachusetts 02115, USA.

[3]Department of Mechanical Engineering, Massachusetts Institute of Technology, Cambridge, Massachusetts 02139, USA.

[4]Division of Plastic Surgery, Department of Surgery, Massachusetts General Hospital, Boston, Massachusetts 02114, USA.

[5]The Hospital for Sick Children, 555 University Avenue, Toronto, Ontario, M5G 1X8, Canada.

[6]Division of Health Sciences & Technology (HST), Massachusetts Institute of Technology, Cambridge, Massachusetts 02139, USA.

† These authors have contributed equally to this work.

* Correspondence to: bvakoc@mgh.harvard.edu

Supplementary Materials

Notes. Anatomy and birefringence of peripheral nerve

Methods I. Animals and histology

Methods II. OCT microscope and data acquisition

Methods III. PS processing for vectorial birefringence analysis

Methods IV. Angiographic processing

Fig. S1. A Schematic of the PS-OCT system.

Fig. S2. A PS-processing flowchart.

Fig. S3. Demyelination of a nerve 5 days after crush injury.

Fig. S4. Comparison of scalar and vector averaging in visualizing myelination.

Fig. S5. Analysis of remyelination following a crush injury.

Fig. S6. A flowchart of motion artifact suppression algorithm.

Fig. S7. Performance analysis of motion artifact suppression.

References

Supplementary Materials:

Notes. Anatomy and birefringence of peripheral nerve

In the peripheral nerve, like in other birefringent biological tissues, the observed birefringence arises from anisotropy both on the molecular level and in the structural arrangement of different constituents. In the axon, microtubules and neurofilaments are contained in the cytoplasm and run in parallel to the longitudinal nerve axis. Schwann cells form a sheath around peripheral axons. This myelin sheath consists of repeating units of lipid bilayers containing membrane proteins. The lipid molecules are oriented in the radial direction, perpendicular to the nerve fiber axis [1,2].

The local birefringence measured by OCT is a result of the combined effect of all microstructures in a voxel. The analysis by Menzel et al. [3] for polarized light imaging of neuronal microstructure and birefringence in the brain provides insights into the birefringence of peripheral nerves. The tubular organelles of the axon cells and the protein framework contribute to a weak positive birefringence with respect to the longitudinal fiber axis. As uniaxial birefringence is defined by the difference in refractive indices of extraordinary and ordinary optic axis, $\Delta n = n_e - n_o$, the slow optic axis is aligned to the nerve fiber's longitudinal axis. In contrast, the anisotropy due to long tails of lipid molecules causes a positive birefringence with respect to the radial fiber axis. Examining the optical properties of a nerve after lipid removal confirmed the lipid in myelin as the dominant source of form birefringence in the nerve [4]. Therefore, the effective birefringence of a nerve fiber with intact myelin is uniaxial negative birefringence with the fast optic axis parallel to the longitudinal axis of nerve fiber. In the displays used in this manuscript, we map the slow optic axis orientation angle θ to our colormap. Such display ensures muscle fiber (having positive uniaxial birefringence) orientation aligns with the hue colorwheel in the en face view images.

Methods I. Animals and histology

Animal protocol

Imaging protocols were performed on the sciatic nerves of transgenic thy1-green fluorescence protein Sprague-Dawley (University of Toronto, Canada) adult rats, weighing 250-350g. The use of the transgenic animal was to explore the option of using fluorescence microscopy to monitor axonal repair in addition to the myelination imaging using optical coherence tomography (OCT). However, the scar tissue and intense vascular remodeling during repair hindered the detection of the fluorescence. The OCT methods described in this study are not affected or dependent on the transgenic modifications.

Anesthesia was achieved with isoflurane (1-3%, maintenance) with a nose cone during both surgical and imaging procedures. The left gluteal hindlimb region was shaved and the skin prepped with betadine. Under aseptic conditions, a 3 cm dorsolateral muscle-splitting incision overlying the left sciatic nerve was made. Using an operating microscope the sciatic nerve was gently dissected from its investing connective tissue exposing it from the sciatic notch to its distal trifurcation; mobilizing the nerve a distance of approximately 20 mm. Using digital calipers, the proximal site injury measured 10 mm from the sciatic nerve trifurcation point. The crush injury group underwent a single crush injury using jeweler's forceps with continuously applied pressure for 30 seconds. The transection and repair with autograft group underwent a transection injury using a #15 blade at 10 mm and 5 mm proximal to the sciatic nerve transection point. This excised segment was reversed and replaced into the 5mm gap to serve as an autograft. The autograft was sutured in place with 10-0 Nylon circumferential epineural sutures. The transection and repair with acellular nerve graft (ANA) underwent a transection injury using a #15 blade at 10 mm and 5 mm proximal to the sciatic nerve transection point. This 5mm gap was repaired with 5 mm acellular nerve grafts which were sutured in place with 10-0 Nylon circumferential epineural sutures. ANA was made of allogeneic nerve scaffolding donated from the sciatic nerves of littermates. These sciatic nerves underwent a chemical decellularization protocol as described by Hudson *et al.* [5]. Following neurorrhaphy repair, the surgical site was closed in layers with 4-0 vicryl and 4-0 monocryl sutures. Topical triple antibiotic ointment

(neomycin/bacitracin/polymyxin) was applied liberally to the closure site. The animals were then returned to a cage and monitored. Once fully recovered from the anesthesia, the animals were returned to the animal facility. The animals were monitored and analgesia (buprenorphine 0.01 mgk/kg IP) was administered twice daily for 72 hours after surgery.

For post-operative imaging sessions, the previously made left gluteal hindlimb incision was prepped with betadine, and the wound was reopened under aseptic conditions. Using an operating microscope, the sciatic nerve was gently exposed from its surrounding scar tissue with care not to disrupt neovascularization for a distance of approximately 20 mm. Imaging was performed on anesthetized animals, and the identical post-imaging procedure as baseline imaging was followed. All animal procedures were approved by the Massachusetts General Hospital Institutional Subcommittee on Research Animal Care and cared for according to the National Institutes of Health Guide for the Care and Use of Laboratory Animals.

Histology

After the last imaging session, animals were euthanized using an injection of pentobarbital (200 mg/kg IP). Following euthanasia, both experimental left and contralateral right sciatic nerves were harvested and processed for histology. For crushed nerve segment, the nerve was harvested from 5 mm proximal to 5 mm distal to the injury site. In the autograft and ANA repairs, the harvest segment was from a distance 5mm proximal to the proximal graft neurorrhaphy site to 5mm distal to the distal neurorrhaphy. Immediately after the harvest the nerve segments were fixed in a 2% glutaraldehyde/2% paraformaldehyde solution. Following 48 hours of fixation, each nerve was cut into proximal, mid-repair and distal sections. Fixed nerves were washed in sodium cacodylate buffer (0.1 M; pH 7.4) and postfixed in 2% osmium tetroxide for 2 hours. After further washing in buffer, specimens were dehydrated in increasing concentrations of ethanol from 25% to 100%. After dehydration, specimens were washed with propylene oxide (Electron Microscopy Sciences) and embedded in epoxy resin consisting of dodecyl succinic anhydrides 98 percent plus free acid 2 percent/tEPON-812 (Electron Microscopy Sciences,

Hatfield, Pa.). Cross-sections (1 μm) were made at each site and stained with 0.5% (w/v) toluidine blue to visualize the nerve microanatomy.

Methods II. OCT microscope and data acquisition

Wide-field and depth-tracking microscope

The benchtop imaging interface was customized specifically for imaging of rodent sciatic nerves. The exposed segment of the nerve was approximately 20 mm in length, along which the nerve follows a curved contour, dictated by the underlying muscle bed. This variation in axial nerve location (Z-dimension) relative to the microscope can be on the order of several to 10 mm. The interface was designed to enable imaging over these fields and across this variable topology while maintaining high transverse resolution for imaging smaller vessels.

As the exposed length of the nerve is larger than the typical field of view achieved by a galvanometer scanner, the full-field scan was accomplished by employing a linear stage (Newport VP-25X) to perform the slow scan by translating the animal along the longitudinal axis of the nerve at a constant velocity. The fast scan was achieved by a galvanometer scanner (Cambridge Technology) placed before the objective lens (Thorlabs LSM03). In this configuration, scanning along the Y- dimension is no longer limited by the objective lens. The maximum transverse field of view was measured to be 10 and 36 mm in the X and Y directions, respectively. Furthermore, the whole microscope including the objective lens and galvanometer was mounted on a motorized stage (Newport VP- 25X) so that the position in the Z-axis could be adjusted to maintain an optimum distance between the objective lens and the sample surface at a given position along the Y-scan dimension. Such additional control helps keeping the sample in focus and maintaining a high transverse resolution throughout the whole imaging volume, especially when the sample has a non-flat surface and the numerical aperture of the objective lens results in a shallow depth of field. In practice, the optimal Z-positions of the microscope are recorded for approximately 10-15 discrete Y-positions prior to each imaging session. A continuous Z-axis profile along Y-scan was then generated using spline interpolation. During imaging, the Z- and Y-axis stages ran synchronously.

Beam scanning protocol

The imaging protocol was set up such that one data volume is collected per session, which was then processed in two independent processing streams for inherently co-registered PS and

angiographic contrasts. While the data collection for PS imaging was facilitated by the electro-optic modulator alternating the polarization state of the probing beam every other A-line, the oversampling for angiography was simultaneously achieved using a bidirectional segmented scan pattern for X-axis. The design was similar to segmented patterns described previously [6-8] but matches forward and backward segments to reduce the required galvanometer bandwidth. The lateral field was divided into four segments. Each point is recorded seven times in total. The galvanometric beam scanner was controlled by a 16-bit digital word streaming from a digital output board (ACCES I/O USB-DO16A) to a digital input module on the galvanometer controller (Cambridge Technology MicroMax series 671XX). To compensate for the delay in the actual beam position relative to the commanded beam position (resulting from the galvanometer control loop latency), we advanced the commanded waveform by 3 samples (0.03 μs) before streaming to the galvanometer controller. The magnitude of this shift was found empirically to best align the forward and backward scan data. For the Y-axis, a motorized stage was used to move the animal at a constant velocity of 60 μm/sec. This scanning induced a displacement of 2 μm between the first and last acquisition of a given segment, which was small relative to the transverse resolution of the system and therefore did not induce significant angiographic noise. The X-axis scan yielded structural and angiographic images with 1184 A-line locations (with each location having 7 measurements). This provided an 8.54 μm X-axis pitch. The frame pitch in the Y-axis direction was 10.56 μm. One full three-dimensional acquisition required 412 seconds.

Methods III. PS processing for vectorial birefringence analysis

Computation of vectorial birefringence

The primary goal of PS processing is to generate a local birefringence vector $\vec{B} = \rho[\cos\theta,\ \sin\theta]$ for each pixel. In this study, the light's polarization state is described using the Stokes formalism [9,10]. Assuming the sample acts as a linear retarder, the local birefringence is described by two parameters: its magnitude is given by the phase retardation ρ [degrees per unit length] and the angle θ that indicates the orientation of the slow optic axis, corresponding to the orientation of the polarization in a plane orthogonal to the beam's propagation direction that experiences the slowest propagation. A flowchart describing the processing scheme is given in Fig. S2.

Note that, in parallel to the PS processing, a standard Fourier-domain OCT processing method [11] was applied to generate a structural intensity tomogram as shown in Fig. S2a. The PS processing starts with background-removed, and chirp-and-dispersion-corrected raw data, which we refer to as signal fringes. The local birefringence parameters (i.e., phase retardation and optic axis) of the tissue was obtained in the Stokes domain using the spectral binning method [12], which was designed to mitigate the effect of polarization mode dispersion in the fiber-based OCT system by calculating the local retardation using several windows of a narrower spectrum. The algorithm computes the local retardation ρ and a three-dimensional retardation eigenvector *(q,u,v)* on the Poincare sphere. In the Stokes formalism, this retardation vector is normalized to have a unit length and provides the orientation of the optic axis.

The retardation vector of a linear retarding element is expected to be confined to the QU-plane, and its orientation angle $\psi = 2\theta$ within this plane corresponds to twice the physical angle of the optic axis. However, birefringence in the imaging system can rotate the observed retardation vectors off the QU-plane [13], as shown in Fig. S2b1. This rotation can be estimated using a least square fitting of the retardation vectors in a region of interest (ROI) defined by the thresholded intensity and retardation. In practice, the fitting was implemented using a principal component analysis on the three-dimensional retardation vectors within the ROI. The least-squares-fitting plane is equivalent to the plane spanned by the first two principal components. Once the plane was found, the data space was transformed to align the fitted plane with the the QU-plane, as shown in Fig. S2b2. Then, all retardation vectors were projected onto the QU-plane, and the

optic axis orientation angle θ was calculated with respect to a manually defined reference angle (marked by the dotted magenta line in Fig. S2b3). This reference angle was determined by the orientation of the fibers in the adjacent muscle tissue that is visible in the en face projection of the structural intensity. We note that preceding tissue layers may also impart a rotation on the retardation vectors of deeper lying tissue when featuring distinct optic axis orientations [14,15]. In the current study, tissues were either homogeneous (muscle), or had orthogonal optic axis orientations (nerves), which cancels the effect of the superficial layer on the subsequent layer. The good quality of the principal component fitting demonstrated in Fig. S2b1 confirms these assumptions.

Lastly, the local birefringence can be visualized with a two-dimensional colormap to show the vectorial nature of PS-contrast. The colormap takes hue and value mapping from the HSV colorspace [16]. The angle of the optic axis orientation ($\theta \in [-90^\circ, 90^\circ]$) was mapped into two revolutions of the hue colorspace using a modulo operation: $\mathrm{H} = \mathrm{mod}[\theta + 90^\circ, 180^\circ] / 180^\circ$. Fig. S3c shows an example frame of angular optic axis orientation colorized with such a colormap. The magnitude ρ, or the amount of phase retardation, was scaled to have a value between 0 to 1 to be mapped to the brightness value and then was multiplied to the intensity mask to exclude the noise dominant pixels. The combined HV colormap of local birefringence is shown in Fig. S2d.

<u>En face projection of vectorial birefringence</u>

A projection based on the alpha compositing [17] can provide microscopic en face perspective of the volume data acquired using the OCT instrument. The HV-mapped vectorial birefringence was converted to its RGB matrix, and each color channel was individually projected to respective XY-transverse planes. A transverse plane is represented by $C[n]$, where C denotes one of the color channels R, G, or B, and the projection index n increases upward starting at n = 1 from the bottom. When projecting the volume data of N planes, the end projection $P_c[N]$ is created using a recursive formula given by:

$$P_c[n] = P_c[n-1](1-\alpha[n]) + C[n]\alpha[n],$$

where the modified opacity $\alpha[n]$ is obtained by multiplying an inclusion mask with the scaled brightness value (V) of each pixel. The inclusion mask was a binary (0,1) mask determined with a threshold on retardance ρ and intensity of a given pixel to exclude the noise-dominant pixels.

Computation of the myelination index (MI)

The formulation of the myelination index (MI) employs this perpendicular orientation to differentiate between the myelin and the epineurium/axons. The MI is based on the angular deviation of the optic axis from the centerline of the nerve. This centerline is manually defined in an en face projection image, providing a distinct centerline angle $\theta_{\mathrm{CL}}[y]$ for every Y-frame. The MI for each pixel is computed using the equation:

$$\mathrm{MI}[x,y,z] = \sum K(x,y,z)\rho[x,y,z]\cos(\theta_{\mathrm{CL}}[y] - \theta[x,y,z]),$$

where *K(x,y,z)* defines a local averaging kernel. The projection images of the MI are generated based on average projections within the fascicular voxels.

We note that the MI is only relevant to the nerve, despite its calculation being applied to the entire volume data including the muscle bed. To avoid confusion, overlay images are generated displaying the colorized MI only within the manually defined nerve ROIs on top of grayscale structural projections, as shown in Fig. S4b and d.

Effects of vectorial treatments

The directionality of the vectorial birefringence should be considered when further processing the acquired PS-OCT measurements. When spatially averaging the data, taking the vectorial mean of birefringence vectors, rather than calculating the scalar mean of local retardance, is critical because the distribution of the optic axis orientation can be drastically different for similar values of local retardation. Therefore, we calculate the mean of vectorial birefringence in the phasor plane as:

$$\mu_v = \left| \sum_q \vec{B}(q) \right| = \left| \sum_q \rho[q] e^{i\theta[q]} \right|.$$

In contrast, the scalar average would be computed using $\mu_s = \sum_q \rho[q]$.

While prior works [18] have demonstrated correlation of birefringence magnitude (i.e., scalar retardance) to histologic myelination, these measures can be improved in accuracy and sensitivity by incorporating the directionality of the birefringence across the three-dimensional datasets. An example for such a case is shown in Fig. S4.

Analysis of remyelination following crush injury

The changes in myelination state following a crush injury can be monitored by analyzing the MI from the vectorial birefringence measurement. A median MI value from a 2 mm segment of the nerve at the crush site was computed for each rat and each timepoint. The average of these MI across animals are presented as a function of time in Fig. S5a. Paired T-test set with a threshold 0.05 indicated significant difference in the longitudinal measurements between post-crush day 14 and 28. A significant difference in MI was observed between day 28 and day 14, consistent with remyelination of the nerve during this timeframe. In Fig. S5b, the MI along the centerline of the common peroneal nerve from the crush location toward the distal end was plotted for all four animals. Representative histology from one animal at day 28 shows evidence of remyelination relative to histology performed at demyelinated timepoints (e.g., Fig. S3).

Methods IV. Angiographic processing

Motion artifact suppression for angiography

For each of the four segments within an X-scan, 21 child-frames are generated based on the 21 angiographic pair combinations of 7 measurements. Each of the child-frames are based on a unique temporal comparison. Our approach is to identify the fraction of these frames that exhibit the lowest bulk motion artifact and use those to generate the final angiographic image, rejecting the other frames affected by motion. To evaluate each angiographic frame according to its motion content, we rank each child-frame according to the ratio of static to dynamic pixels. The underlying assumption is that only the static pixels are vulnerable to bulk motion, because the measurement interval is large enough to give a completely decorrelated signal for the blood flow. Next, we chose *nP* frames (in the work, a value of 5 was empirically chosen for *nP*) with the highest ratio of static to dynamic pixels. The remaining frames are discarded. The selected frames are then averaged to generate the final angiographic frame. This averaging is performed according to the equation:

$$f_{CDV}(z) = \sqrt{1 - \frac{\left|\sum_{t=1}^{nP}\sum_{k=-L}^{L} w(k)R(z-k,t)R^{*}(z-k,t+1)\right|}{\sum_{t=1}^{nP}\sum_{k=-L}^{L} w(k)\frac{1}{2}\left[|R(z-k,t)|^{2} + |R(z-k,t+1)|^{2}\right]}} ,$$

where averaging is performed in the numerator and denominator rather than across the ratios. Here, R is the complex OCT signal, A is the amplitude (i.e., $|R|$), and w is a windowing kernel. In this work we use a Hann kernel of size 11. Prior works demonstrated that averaging in the numerator and denominator yields better performance and noise rejection [19]. After all frames are processed, a short sliding window minimum filter was applied in the Y-dimension to further reduce the motion artifact.

Fig. S7 demonstrates the reduction in motion achieved by the aforementioned method. The severe phase washout in the vascular contrast tomogram and the white lines in the transverse slice are prominent in Fig. S7a. The equivalent tomogram and en face projection after the motion artifact rejection are shown in Fig. S7b. The apparent reduction of the motion artifact and

improved contrast is confirmed by the additional plots. The histogram of ROI a (green rectangle) is displayed in Fig. S6c1, where the bimodal histogram confirms the contrast between the vessel and static regions. Fig. S7c2 shows the line profile of ROI b (green line) where the maximum pixel value decreased from 191 to 63.

Segmented angiography projection

The high contrast of epineurium provided by its vectorial birefringence allows for simple manual segmentation of the intra-fascicular voxels. We create a binary green-pseudocolor mask for the fascicle and use it for the en face projection. The projection is created using the alpha compositing method similar to the vectorial birefringence projection. The difference in this application is that the inclusion mask is generated using the structural intensity data to suppress the contribution of pixels with low SNR. An angiographic processing toolbox including this projection algorithm is available online (http://octresearch.org/resources/downloads/).

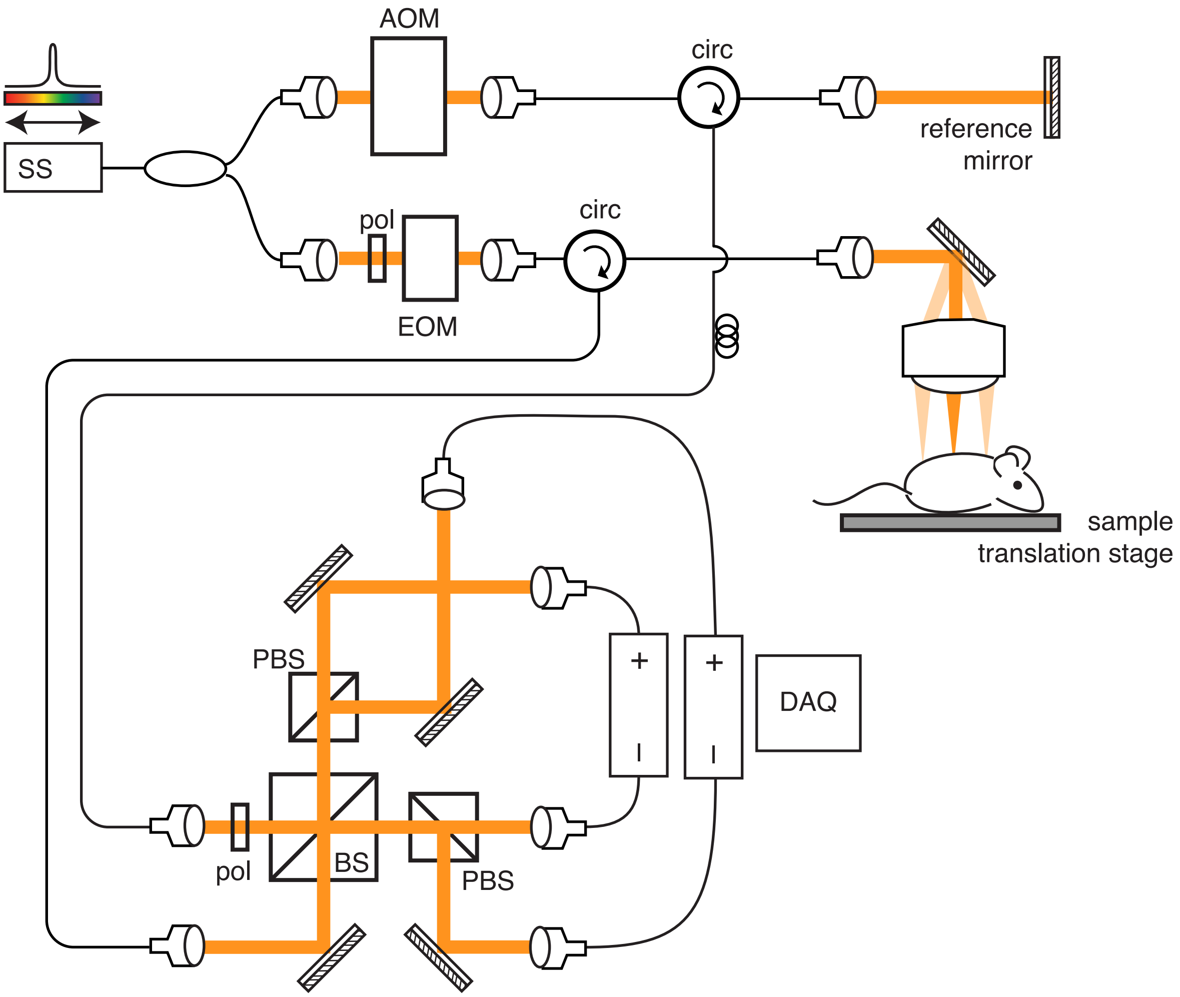

Fig. S1. A Schematic of the PS-OCT system. SS: swept source, AOM: acousto-optic modulator, pol: polarizer, EOM: electro-optic modulator, circ: circulator, PBS: polarizing beam splitter, BS: beam-splitter (non-polarizing)

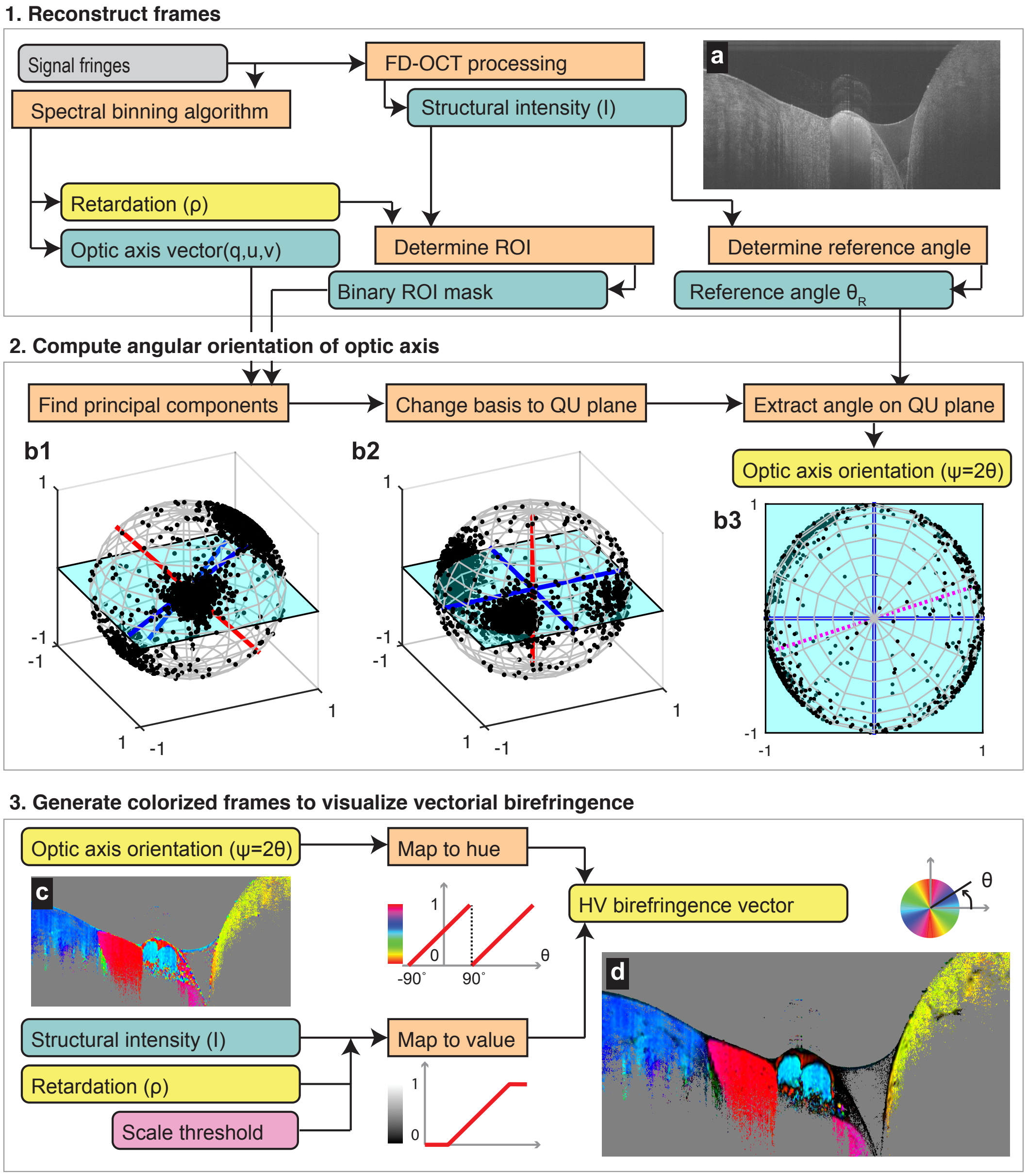

Fig. S2. A PS-processing flowchart. a, A structural intensity tomogram (conventional OCT). b, The Poincaré sphere representation illustrating the computation of the optic axis angle θ. b1, All black dots on the Poincaré sphere represent the optic axis vectors *(q,u,v)* of individual pixels within the ROI. Blue axes are the first and second principal components (PC) of the retardation eigenvectors. b2, The optic axis vector space is transformed so the new QU-plane spans the first two PCs of the transformed optic axis vectors. b3, The transformed optic axis vectors are projected onto the QU-plane, and the relative angle of each vector with respect to the pre-defined reference is computed. c, the angular optic axis orientation visualized with the hue mapping. d, Visualization of the vectorial birefringence in HV color space.

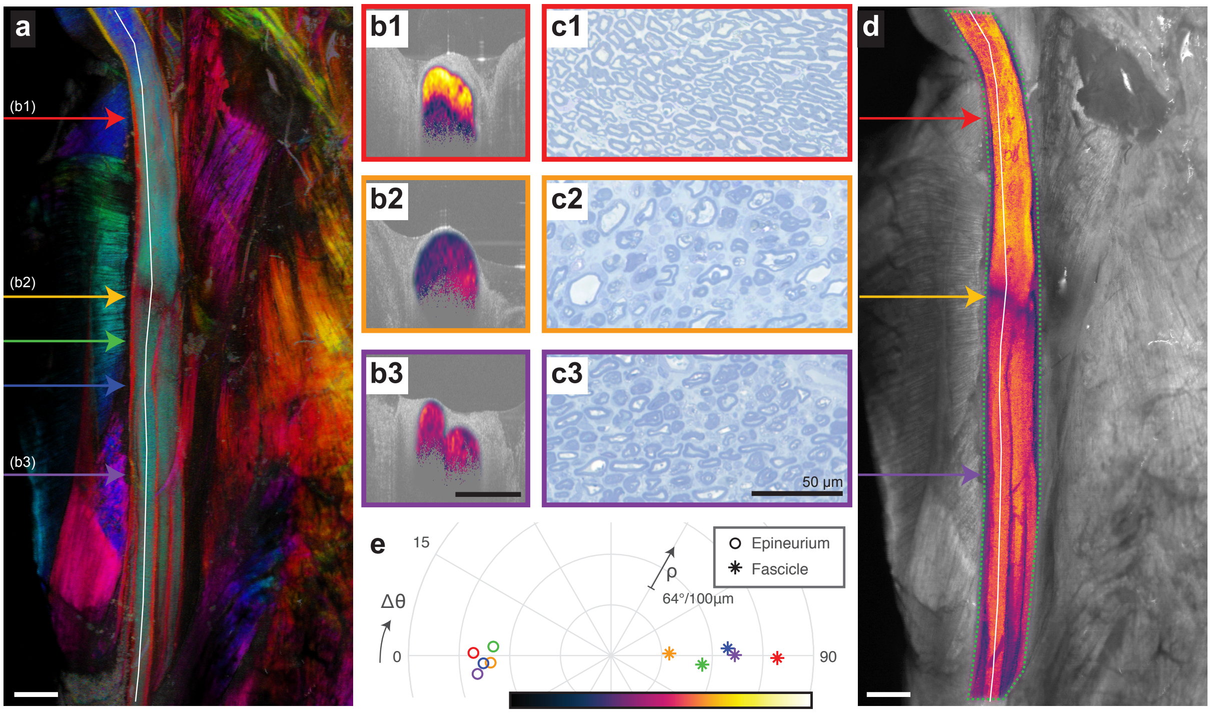

Fig. S3. Demyelination of a nerve 5 days after crush injury. a, Wide-field projection of vectorial birefringence. Colored arrows denote the cross-sections used to perform the vectorial birefringence analysis of epineurium and fascicle as shown in the polar plot of (e). b, Cross-sectional visualization of myelination index. The cross-sections (1-3) correspond to the locations marked by red, yellow and purple arrows. c, Corresponding histology. d, Overlay of MI projection on the structural projection. e, Polar plot of vectorial birefringence in manually determined ROIs of epineurium and fascicle.

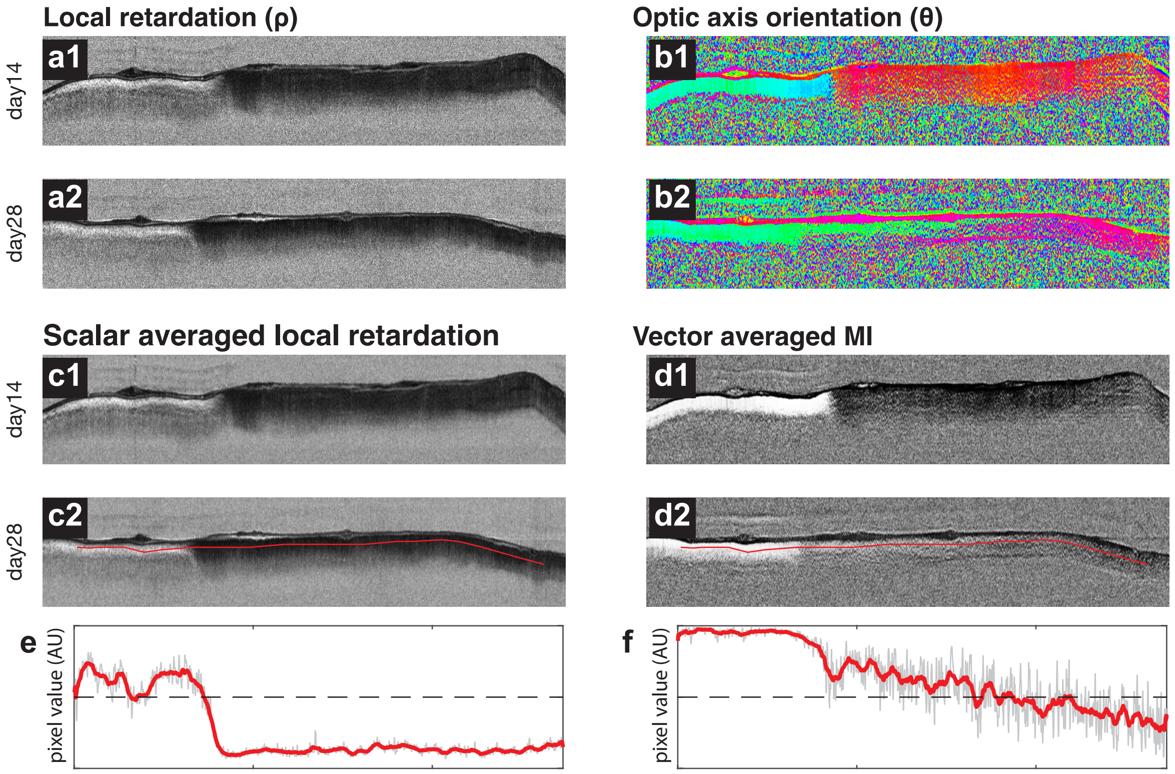

Fig. S4. Comparison of scalar and vector averaging in visualizing myelination. Cross-sections along the centerline on days 14 and 28 after a crush injury, from top (1) to bottom (2). a, Local retardation: the low values of birefringence magnitude is apparent in both timepoints as darker pixels. b, optic axis orientation: despite small retardation, two timepoints show striking difference in fascicular optic axis orientation. The optic axis orientation correctly captures the remyelination on the post-repair day 28. c-d, scalar and vector average of local retardation. e-f, Pixel value plots along the centerlines marked in (c2 and d2), respectively. The high sensitive of optic axis orientation in measuring myelination translates to spatial averaging, where the contrast for remyelination is only provided by vectorial averaging.

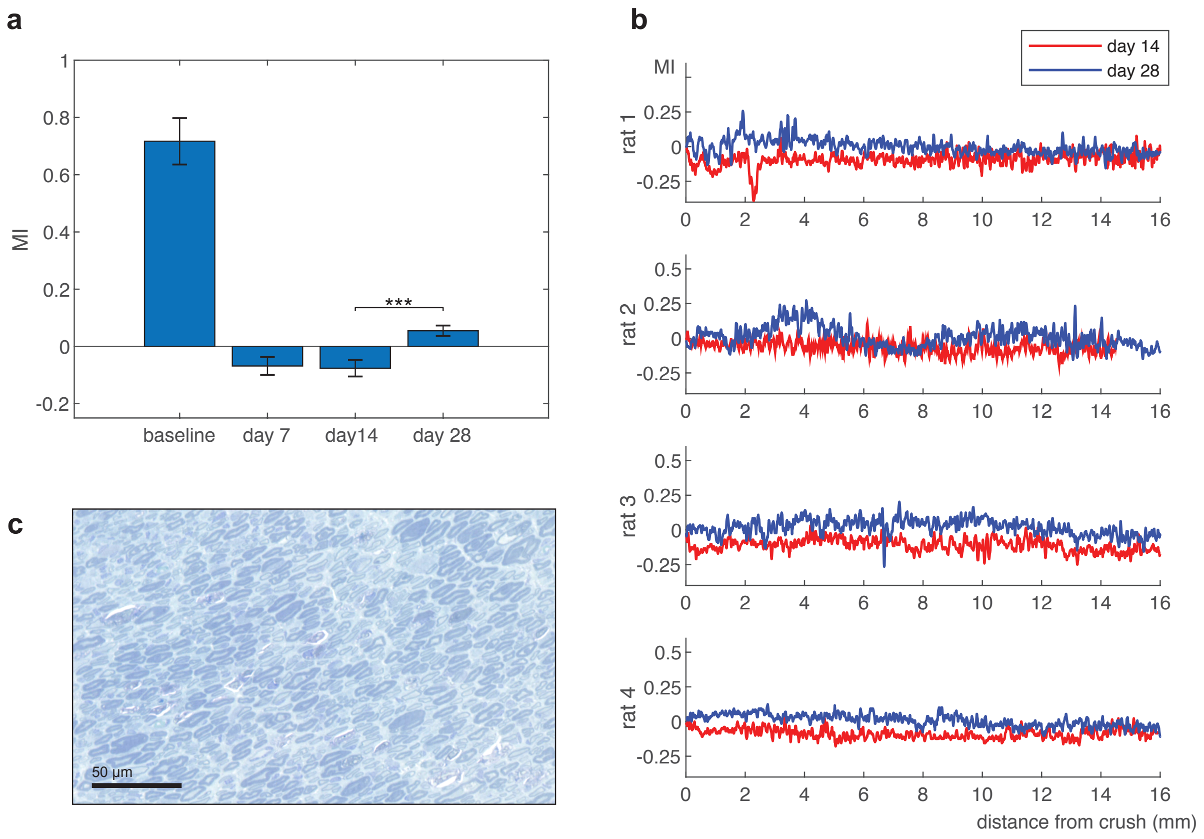

Fig. S5. Analysis of remyelination following a crush injury. a, analysis of longitudinal changes in MI in the crush group. A p-value of 0.0005 was computed using a paired T-test between the MI measurements of days 14 and 28. b, Plots of MI in the nerve 14 days (red) and 28 days (blue) after the injury, displayed for all animals that were subject to crush injury. The values are plotted along the centerline of the common peroneal nerve as a function of the distance distal to the crush site (distance = 0). c, Histology of the crush site at 28 days post-crush.

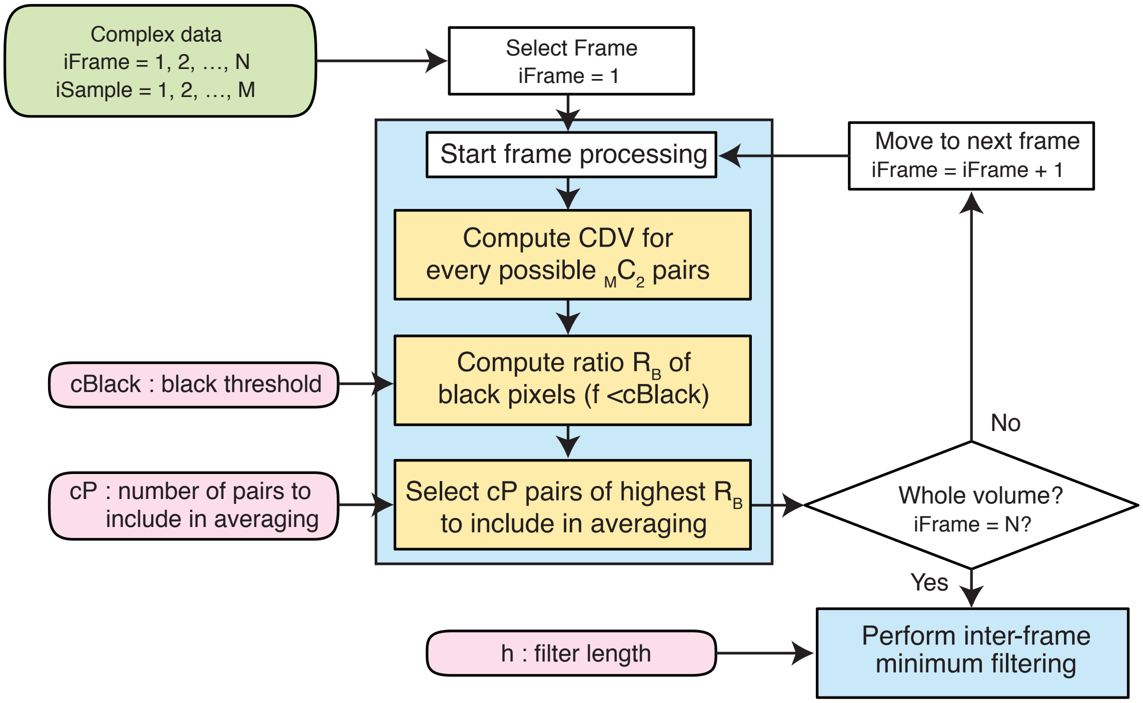

Fig. S6. A flowchart of motion artifact suppression algorithm.

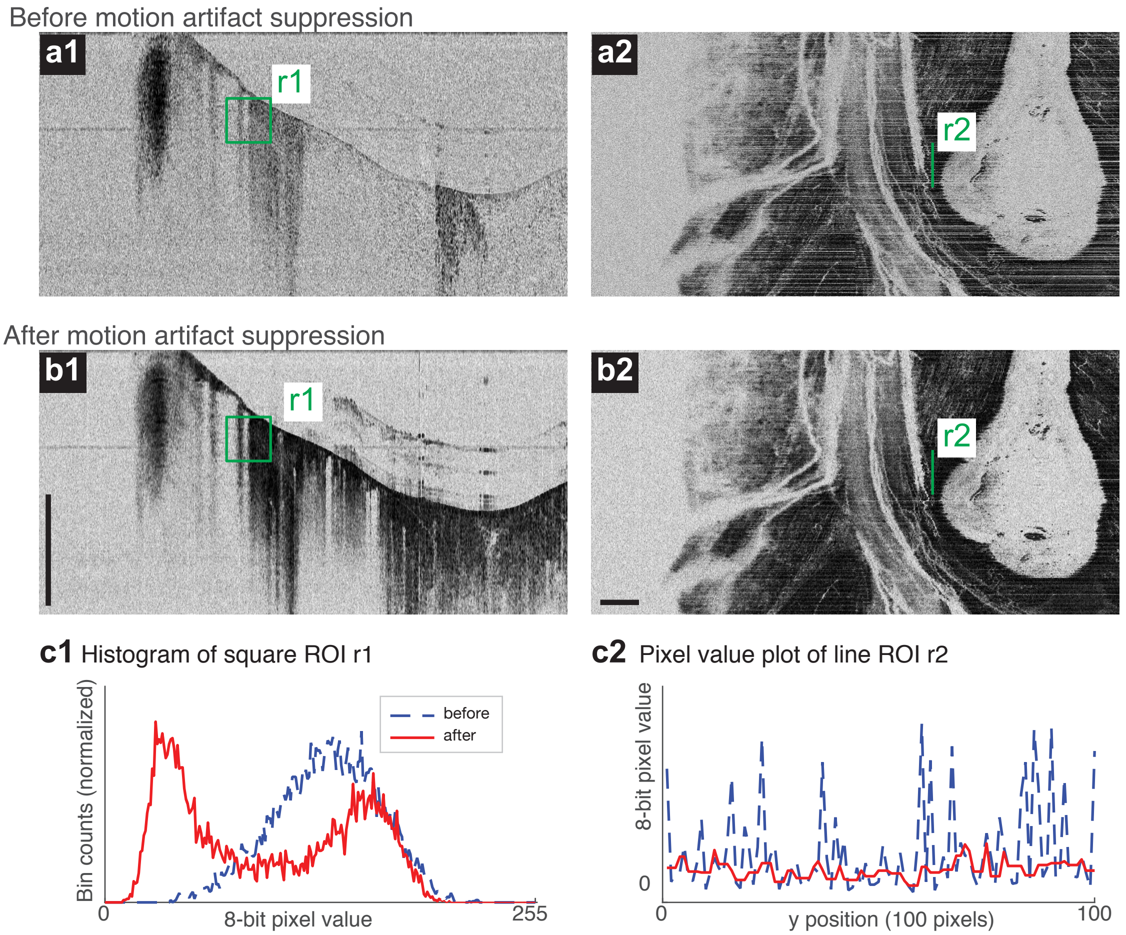

Fig. S7. Performance analysis of the motion artifact suppression. a, Angiographic tomogram (a1) and en face (a2) angiography before the motion suppression. b, Respective tomogram and en face angiography after the algorithm is applied. c1, Histogram of angiographic pixel values of square ROI r1, marked with the green squares in a1 and b1. c2, A plot of vascular contrast pixel values in the line ROI r2, marked with the green lines in a2 and b2.

References

1. Min, Y. *et al.* Interaction forces and adhesion of supported myelin lipid bilayers modulated by myelin basic protein. *Proc. Natl. Acad. Sci. U.S.A.* **106,** 3154–3159 (2009).
2. Côté, D. *et al.* Quantitative myelin imaging with coherent anti-Stokes Raman scattering microscopy: alleviating the excitation polarization dependence with circularly polarized laser beams. *Opt Express* **17,** 18419–18432 (2009).
3. Menzel, M. *et al.* A Jones matrix formalism for simulating three-dimensional polarized light imaging of brain tissue. *J. R. Soc. Interface* **12,** 20150734–14 (2015).
4. de Campos Vidal, B., Mello, M. L. S., Caseiro-Filho, A. C. & Godo, C. Anisotropic properties of the myelin sheath. *Acta Histochemica* **66,** 32–39 (1980).
5. Hudson, T. W. *et al.* Optimized acellular nerve graft is immunologically tolerated and supports regeneration. *Tissue Eng.* **10,** 1641–1651 (2004).
6. Vakoc, B. J., Lanning, R. M., Tyrrell, J. A. & Padera, T. P. Three-dimensional microscopy of the tumor microenvironment in vivo using optical frequency domain imaging. *Nature Medicine* (2009). doi:doi: 10.1038/nm.1971
7. Grulkowski, I. *et al.* Scanning protocols dedicated to smart velocity ranging in spectral OCT. *Opt Express* **17,** 23736–23754 (2009).
8. Braaf, B., Vermeer, K. A., Vienola, K. V. & de Boer, J. F. Angiography of the retina and the choroid with phase-resolved OCT using interval-optimized backstitched B-scans. *Opt Express* **20,** 20516–20534 (2012).
9. de Boer, J. F. & Milner, T. E. Review of polarization sensitive optical coherence tomography and Stokes vector determination. *J Biomed Opt* **7,** 359–371 (2002).
10. Park, B. H., Pierce, M. C., Cense, B. & de Boer, J. F. Real-time multi-functional optical coherence tomography. *Opt Express* **11,** 782–793 (2003).
11. Yun, S. H., Tearney, G. J., de Boer, J. F. & Bouma, B. E. Removing the depth-degeneracy in optical frequency domain imaging with frequency shifting. *Opt Express* **12,** 4822–4828 (2004).
12. Villiger, M. *et al.* Spectral binning for mitigation of polarization mode dispersion artifacts in catheter-based optical frequency domain imaging. *Opt Express* **21,** 16353–16369 (2013).
13. Park, B. H., Pierce, M. C., Cense, B. & de Boer, J. F. Optic axis determination accuracy for fiber-based polarization-sensitive optical coherence tomography. *Opt. Lett.* **30,** 2587–2589 (2005).
14. Fan, C. Mapping local optical axis in birefringent samples using polarization-sensitive optical coherence tomography. *J. Biomed. Opt.* **17,** 110501–110501 (2012).
15. Fan, C. & Yao, G. Imaging myocardial fiber orientation using polarization sensitive optical coherence tomography. *Biomed. Opt. Express, BOE* **4,** 460–465 (2013).
16. Joblove, G. H. & Greenberg, D. Color spaces for computer graphics. *ACM siggraph computer graphics* (1978).
17. Porter, T. & Duff, T. Compositing Digital Images. *SIGGRAPH Comput. Graph.* **18,** 253–259 (1984).
18. Henry, F. P. *et al.* In vivo optical microscopy of peripheral nerve myelination with polarization sensitive-optical coherence tomography. *J. Biomed. Opt.* **20,** 046002–046002 (2015).
19. Nam, A. S., Chico-Calero, I. & Vakoc, B. J. Complex differential variance algorithm for optical coherence tomography angiography. *Biomed. Opt. Express, BOE* **5,** 3822–3832 (2014).
